# Respiratory tract lining fluid copper content contributes to pulmonary oxidative stress in patients with systemic sclerosis

**DOI:** 10.12688/wellcomeopenres.20080.2

**Published:** 2025-03-04

**Authors:** Andreas Frølich, Rosamund E. Dove, Maria Friberg, Annelie F. Behndig, Thomas Sandström, Anders Blomberg, Ian S. Mudway

**Affiliations:** 1 Department of Public Health and Clinical Medicine, Umeå University, Umeå, Sweden; 2 Wolfson Institute of Population Health, Queen Mary University of London, London, England, UK; 3 MRC Centre for Environment and Health, Environmental Research Group, Imperial College London, London, England, UK

**Keywords:** Systemic sclerosis, fibrosis, oxidative stress, copper, respiratory tract lining fluid, chronic lung disease, interstitial lung disease, bronchoalveolar lavage

## Abstract

**Background:**

Systemic sclerosis (SSc) is an autoimmune disease characterized by fibrosis of the skin and internal organs, mostly affecting young and middle-aged women. Significant questions remain as to its pathogenesis, especially the triggers for the associated interstitial lung disease (SSc-ILD). We examined the extent to which SSc and SSc-ILD were related to oxidative stress and altered metal homeostasis at the air-lung interface.

**Methods:**

In this case-control study, we recruited 20 SSc patients, of which 11 had SSc-ILD. Eighteen healthy individuals were recruited as age-matched healthy controls, for a total of 38 study participants. Low molecular weight antioxidants (ascorbate, urate and glutathione), metal transport and chelation proteins (transferrin and ferritin) and metals (Fe and Cu) concentrations, including a measure of the catalytically active metal pool, were determined in respiratory tract lining fluid (RTLF) collected by bronchoalveolar lavage from the SSc group and compared with healthy controls.

**Results:**

In the SSc group, 14 individuals were of female sex (70%) and the median age was 57 years (range 35–75). We observed evidence of oxidative stress in the RTLFs of SSc patients, characterised by increased concentrations of glutathione disulphide (GSSG, P<0.01), dehydroascorbate (DHA, P<0.05) and urate (P<0.01). This was associated with elevated RTLF Fe (P=0.07) and Cu (P<0.001), and evidence of a catalytic metal pool, demonstrated by an enhanced rate of ascorbate oxidation in the recovered lavage fluid (p<0.01). Cu concentrations were significantly associated with the ascorbate depletion rate (r=0.76, P<0.001), and GSSG (r=0.38, P<0.05) and protein carbonyl (r=0.44, P<0.01) concentrations. Whilst these markers were all increased in SSc patients, we found no evidence for an association with SSc-ILD.

**Conclusions:**

These data confirm the presence of oxidative stress in the airways of SSc patients and, for the first time, suggest that an underlying defect in metal homeostasis at the air-lung interface may play a role in disease progression.

## Plain language summary

Systemic sclerosis (SSc) is an autoimmune disease affecting the skin and inner organs. It usually affects young and middle-aged women and the disease process is still not fully understood. Some patients with SSc go on to develop the associated interstitial lung disease (SSc-ILD), which carries with it a high mortality. It is important that these patients be diagnosed correctly and receive treatment an early stage of the disease. What is currently established is that these patients have high levels of oxidative stress and we are investigating whether trace metals play a role in the disease process of both SSc and SSc-ILD. In addition to improving our understanding of this disease, we aim to improve our diagnostic options as well as treatment, as medications that bind trace metals are readily available and safe.

## Introduction

Systemic sclerosis (SSc) is an autoimmune disease characterised by progressive fibrosis of the skin and/or the internal organs, vasculopathy and autoantibodies against various cellular antigens. It is a rare condition with a heterogeneous range of clinical manifestations, mostly affecting young and middle-aged women ^1^. SSc can be divided into two main types; one limited type affecting the skin and one diffuse type.

Systemic sclerosis-associated interstitial lung disease (SSc-ILD) is a common manifestation in SSc. SSc-ILD can lead to progressive lung fibrosis and remains the main cause of morbidity and mortality in this patient group, with a prevalence of up to 30% and a 10-year mortality of up to 40% ^2^. Previous studies have reported sex differences in both prevalence and clinical outcomes in SSc-ILD, with male sex being associated with the presence of SSc-ILD as well as an increased risk of SSC-ILD disease progression ^2^. There is also some geographical variation in both overall prevalence and sex distribution in SSC-ILD ^3^. With regard to the observed sex differences, social factors such as variable occupational exposures and disparities in health-care system utilisation have been proposed as possible explanations ^4^. There is also some evidence to support the possibility of a biological difference in the treatment effect between sexes ^4^.

Due to a lack of awareness of the typical signs or symptoms of SSc and SSc-ILD, patients are often diagnosed only during the later stages of the condition, when the disease has already progressed to a more severe pulmonary fibrosis. These circumstances have been reflected upon in the recent revisions made in the EULAR classification, in order to improve the detection of early-stage disease and/or limited cutaneous types of the disease ^5^.

There is an acknowledged unmet need for reliable peripheral blood and/or bronchoalveolar lavage (BAL) to improve the clinical diagnostic work-up of patients with SSc-ILD for clinicians, as well as a need to elucidate the disease mechanisms, which remain poorly understood. As shown in a recent meta-analysis reviewing the usefulness of BAL in monitoring disease activity in SSc, the presence of alveolitis and increased levels of inflammatory markers in BAL, including tumor necrosis factor alpha (TNF-α), interleukin 6 (IL-6), interleukin 7 (IL-7) and interleukin 8 (IL-8), were associated with impaired lung function, increased symptoms and/or worse radiological features ^6^. When it comes to SSc, several potential candidate biomarkers have been identified in peripheral blood and/or BAL, including the glycoprotein Krebs von den Lungen-6 (KL-6), surfactant protein D (SP-D) and IL-8, but further validation of these is warranted ^7^. 

A key feature of SSc pathogenesis is chronic oxidative stress, where elevated levels of reactive oxygen species (ROS), through an imbalance between oxidant and anti-oxidant states, impacts inflammation, fibrosis and autoimmunity ^8, 9^. Levels of several blood biomarkers of oxidative stress, including nitric oxide and malondialdehyde, have consistently been higher in patients with SSc when compared to controls. Additionally, the antioxidant markers vitamin C and superoxide dismutase have been found to be lower in SSc patients than in controls ^10^. The current understanding of the aetiology of SSc-ILD remains incomplete but likely involves a wide range of interconnected adverse biological responses within and beyond the lungs. A triggering event in the disease has of yet not been identified, but it is likely an autoimmune process against mesenchymal cells ^1^. The presence of alveolitis, typically characterised by increased numbers of activated macrophages, polymorphonuclear cells (PMN), eosinophils and, occasionally, lymphocytes, has for a long time been viewed as a first step in the development of SSc-ILD ^11^. Activated macrophages are the primary pulmonary immune mediator cells, secreting cytokines, including tumor necrosis factor alpha (TNF-a), interleukin 6 (IL-6), and interleukin 8 (IL-8). TNF-a is a proinflammatory fibrogenic cytokine that appears early in the inflammatory response and has adverse impacts on endothelial cells ^11^. A consistent finding is evidence of increased ROS generation at very early stages of the disease, leading to increased levels of systemic oxidative stress, that coincide with the development of vascular dysfunction, perivascular inflammation and pulmonary inflammation ^1, 9^. The interplay between vascular injury, inflammation, altered tissue repair, coagulability and fibrinolysis leads to a more inflammatory environment and the release of profibrotic stimuli at the cellular level ^2^. Genetic background also appears to play a role, supported by previous observations of higher incidence of SSc in relatives of patients with SSc than in the general population as well as an increased incidence in certain ethnic groups ^12^.

While ROS can exhibit a number of adverse biological effects, they are not generally damaging at physiological concentrations, due to the presence of comprehensive antioxidant defenses. However, their reactions with poorly liganded metal species, such as iron and copper, can lead to the catalytic production of the very reactive hydroxyl radical ( ^•^OH), which is exceptionally damaging ^13^. Excess iron concentrations in the body have been associated with the pathogenesis of abnormal ageing, neurodegenerative disease and cancer ^14, 15^, presumably via the generation of ROS. Excess iron accumulation in the lung has been reported in association with cigarette smoke ^16– 18^ and severe emphysema ^19^. In a 2014 study, Philippot and colleagues identified the alveolar macrophage as the predominant iron-positive cell type in lung tissues ^20^, with the quantity of iron deposits and the percentage of iron-positive macrophages increasing with chronic obstructive pulmonary disease (COPD) and emphysema severity, suggesting that there is an activation of an iron sequestration mechanism by alveolar macrophages in COPD as a protective mechanism against iron-induced oxidative stress. Little is however known about iron accumulation in SSc and SSc-ILD.

Whilst copper is an essential component of cytoplasmic superoxide dismutase (SOD), elevated levels of copper, both in serum and tumor tissue, have been reported in a variety of malignancies and in relation to cancer progression ^21^. Less is known about the relationship between of copper and the pathophysiology of both SSC and SSC-ILD, but some observational data point to altered levels of copper in SSc patients, possibly through mechanisms of increased oxidative stress ^22^. To our knowledge, no data are available on the role of catalytic metals in the airways of patients with SSc and/or SSc-ILD. Looking outside of the airways, available data are diverging on whether systemic copper dysregulation is present in this patient group, as shown in a study by Hughes and colleagues showing that plasma copper levels in SSc patients were not increased when compared to controls ^23^.

As to SSc-ILD, available data from other pulmonary diseases suggests that BAL iron and copper levels are increased in patients with idiopathic pulmonary fibrosis (IPF) compared to controls ^24^. This study investigated the hypothesis that oxidative stress and redox active metals (iron and copper) at the air-lung interface are features of SSc, specifically contributing to SSC-ILD.

## Methods

### Study design and setting

The present study used a case-control design and included a sample of patients with SSc (n=20) as the diseased group. At the time of recruitment this represented all SSc patients undergoing evaluation and treatment at the Department of Rheumatology at the University Hospital of Umeå, Umeå, Sweden. The study size was not pre-determined, rather it reflected the number of patients with SSc that were available to us. All 20 patients were contacted and invited to join the study, and all of them accepted to participate (100%). All of the participants had been diagnosed by an experienced rheumatologist using the 1980 ARA criteria. The participants were given both oral and written information about the study. Sex was self-reported for both groups. As the diseased group represented the entire population of SSc patients that were treated at our hospital, any further exclusion to this group was not considered. The healthy controls were selected to achieve age and sex matching with the diseased group. A history of asthma and/or allergy were the main exclusion criteria for the healthy controls.

All procedures were undertaken by trained medical staff at the University Hospital, Umeå, Sweden. Data collection by bronchoscopy was performed from November 1995 to January 2003.

The delay between data collection and publication is explained by the current methods of metal and antioxidant analysis having been added to the study methods only after all of the BAL samples had been collected. The current research aims were subsequently revised, reflecting this change in study methodology.

Study approval was granted by the local ethics committee at Umeå University (Um dnr 95–303) in November 1995 and the study was performed according to the principles of the Declaration of Helsinki, with written informed consent obtained from all study participants prior to enrolment.

### Subjects and bronchoscopies

In the present study, 20 patients with SSc (mean age with range 57 (35–75) years, 14 female), 11 with SSc-ILD (four smokers and six on oral corticosteroid therapy), confirmed by high resolution computed tomography (HRCT) (Philips Tomoscan LX, Single slice scanner, MA, USA), and 18 age-matched controls (57 (38–70) years, 14 female) underwent routine fibre-optic bronchoscopy (Olympus BF T10 or T20, Olympus. Tokyo, Japan) with bronchoalveolar lavage (BAL) to sample peripheral RTLFs. Full details of subject demographics and clinical parameters within the SSc patients are presented in Table 1. All SSc patients underwent baseline lung function assessment (lung volumes, dynamic spirometry and CO diffusion capacity measurement (Master spirometer and Master Pro-Transfer; Jaeger, Würzburg, Germany) prior to bronchoscopy – Table 1.

**Table 1.** Control subject and patient characteristics.

Characteristics	Healthy controls (n=18)	SSc patients (n=20)	SSc without ILD on HRCT (n=9)	SSc with ILD on HRCT (n=11)	P-value
**Age** (mean, range), years	57 (38–70)	57 (35–75)	49 (35–64)	64 (47–75)	0.002 ^a^
**Sex** (M/F)	4/14	6/14	1/8	5/6	
**Disease duration** (mean, range), years	-	9.7 (1.0-35.0)	14.7 (3.0-35.0)	5.6 (1.0-18.0)	0.03 ^a^
**Limited / diffuse SSc**	-	16/4	8/1	8/3	
**Auto-antibodies** ACA ( *anti-centromere*), ANA ( *anti-nuclear*), RNP ( *anti-ribonucleoprotein*), Scl-70 ( *scleroderma-70*), SSA ( *Sjögren's syndrome A antigen*), SSB ( *Sjögren's * *syndrome B antigen*).	-	ACA (10), RNP (3), ANA (4), Scl- 70 (3), SSA (2), SSB (2)	ACA (6), RNP (1), ANA (2)	ACA (4), RNP (2), ANA (2), Scl-70 (3), SSA (2), SSB (2)	
**Sjögren's syndrome**	0	6	1	5	
**Smokers**	0	4	2	2	
**Oral corticosteroid treated**	0	6	2	4	
**VC** (median, 25–75 ^th^ percentiles), % of predicted	-	104 (96–122)	120 (100–124)	101 (85–116)	NS ^b^
**TLC** (median, 25–75 ^th^ percentiles), % of predicted	-	100 (89–114)	114 (98–121)	91 (88–111)	0.007 ^b^
**FEV _1_** (median, 25–75 ^th^ percentiles), % of predicted	-	95 (87–104)	97 (90–104)	90 (82–106)	NS ^b^
**DL _CO_** (median, 25–75 ^th^ percentiles), % of predicted	-	99 (94–103)	100 (93–102)	96 (90–107)	NS ^b^

VC= vital capacity; TLC = total lung capacity; FEV _1_ = forced expiratory volume during the 1 ^st^ second; DL _CO_ = diffusion capacity for carbon monoxide. SSc with and without ILD confirmed by high-resolution computed tomography (HRCT). ^a^Comparison between age and disease duration in SSc patients was performed using an unpaired T-test; ^b^Comparisons between lung function parameters were performed using the Mann-Whitney U test

Methods and protocols for BAL sample treatment, antioxidant and metal analysis are available as a collection in protocols.io https://dx.doi.org/10.17504/protocols.io.ewov1qmxygr2/v1

Bronchoscopies with BAL were performed with subjects in the supine position, with three separate instillations of 60 mL of sterile phosphate buffered saline (PBS) (pH 7.3, 30°C) into a segmental bronchus of the middle lobe of the right lung. The recovered aspirates from each separate instillation were pooled, passed through a sterile nylon filter to remove mucus (pore diameter 100 μm, Syntab Product AS, Malmö, Sweden) and centrifuged at 400 g for 15 minutes (4°C) to pellet airway cells. The cell free supernatants were then aliquoted for storage at -80°C until required for analysis. Details on the differential cell counts and soluble mediator concentrations in these groups have been published previously ^25^. Earlier studies have examined the procedural factors that may be significant in causing variability in the concentration of lavage components, such as dwell time, the volume of saline instilled, the number of aliquots and the inclusion or exclusion of the first lavage instillment ^26^. There is some inter-centre variability in how the procedure is performed and which dilution methods are used, and recent guidelines on flexible bronchoscopy have been published with the aim of standardizing the procedure ^27^. We used the same procedural steps as in previous studies by our research group, where a more detailed method description is provided ^28^.

### Antioxidant and metal analysis

The lavage returns were analysed for low molecular weight antioxidants (ascorbate [AA], urate – [UA] and glutathione [GSH]), their oxidation products (glutathione disulphide [GSSG] and dehydroascorbic acid [DHA]), as well as the Fe transport and chelation proteins transferrin and ferritin. Briefly, BAL fluid GSH concentrations were determined using the glutathione disulphide (GSSG)-reductase-dithiobis-2-nitrobenzoic acid (DTNB) recycling method ^29^. BAL fluid ascorbate (AA) and urate (UA) were measured by reverse phase HPLC with electrochemical detection in samples following lipid extraction with heptane and acidified with metaphosphoric acid ^30^. Total vitamin C [dehydroascorbate (DHA)+AA] was determined by pre-treating acidified samples with the reductant 50 mM Tris(2-carboxylethyl)phosphine (TCEP) (Molecular Probes, Eugene, OR, USA) for 15 min prior to HPLC analysis. The DHA concentration was calculated by subtracting the measured ascorbate from the total vitamin C concentration, as previously described ^25^. Human transferrin and ferritin (undiluted) were quantified in BAL fluid samples using ELISA-kits from Alpha Diagnostic International, Inc., San Antonio, TX, USA, according to the manufacturer’s instructions. Total protein concentrations in the lavage fluids were determined using the bicinchoninic acid assay, with protein carbonyl levels quantified following derivatization with 2,4-dinitrophenylhydrazine with a commercial immunoassay (ALX-850-312-K101, Alexis Biochemicals AXXORA, LLC, San Diego, CA, USA) ^31^. Protein carbonyl concentrations were expressed per milligram of protein.

Lavage fluid total Fe and Cu concentrations were determined by ICP-MS following microwave digestion (30 min at 1600W - CEM Mars 5 Digestion Oven): 375 µL of lavage fluid added to 1,125 µL of 6.5% HNO _3_ and spiked with 1 ppb Yttrium (final concentration) as an internal standard (IS). Samples were analysed for ^56^Fe and ^63^Cu ICP-MS (ELAN DRC, MSF008), in DRC mode, employing ammonia at a flow rate 0.7 mL/min to remove potential isotopic interferences. Elemental concentrations were determined with reference to a 6-point standard curve based on an ICP multi-element standard solution VI CertiPUR® (Merck, Lot. No. OC529648). All final concentrations were calculated following subtracted on the digestion blank; PBS taken through the whole digestion protocol, and correction for the IS recovery across prescribed mass ranges.

The pro-oxidant activity of the BAL fluid samples was determined by following their capacity to deplete exogenous ascorbate (AA) added to the samples to achieve an initial starting concentration of 200 μM. A stock AA solution was prepared at a concentration of 4 mM in Chelex-100 resin treated ultra-pure (18 Ohm resistivity) water and adjusted to pH 7.0. An aliquot of each lavage sample (90 μL) was then diluted with 5 μL of Chelex-treated water and then incubated with the stock antioxidant solution (5 μL) at 37°C for two hours in a plate reader (Spectra Max 190). Lavage fluid incubations with AA were performed in triplicate in UV 96-well flat-bottom plates (Greiner bio-one). The concentration of AA remaining in each well was quantified by measuring the absorbance at 265 nm every two minutes over the two-hour incubation period. Duplicate blanks and standards (25–200 μM AA) were run in parallel with samples on the 96-well plate, such that a calibration curve was constructed for each two-minute measurement. The AA concentration in sample wells at each time point was determined against its respective calibration curve and corrected for AA losses due to auto-oxidation measured in the blank controls. To determine the influence of metals in the measured rate of AA depletion, samples were incubated with the metal cation chelators diethylene triamine pentaacetic acid (DTPA) and nitrilotriacetate (NTA). Incubations were conducted in a similar procedure as described above, however, instead of diluting the samples with 5 μL Chelex-treated water, samples were spiked with (5 μL) or either 4 mM DTPA or NTA (200 μM final concentration), prior to the addition of AA.

### Statistical analysis

Lavage fluid data and lung function parameters were not normally distributed and are therefore expressed throughout as median concentrations, with 25 ^th^ and 75 ^th^ percentiles. Comparisons between control and disease groups, including between SSc patients with and without HRCT-confirmed pulmonary fibrosis were performed using the Kruskal-Wallis one-way ANOVA, with post-hoc testing performed using the Mann-Whitney U test. Associations between endpoints were evaluated using the Spearman rank order correlation test. All statistical analysis were performed using IBM SPSS Statistics (RRID:SCR_016479) v. 28 and, in all cases, significance was assumed at the 5% level.

## Results

The 11 patients with SSc with associated ILD, as confirmed by HRCT, displayed significantly reduced total lung capacity compared to the patients without ILD ( Table 1), characteristic of restrictive airway disease. All other spirometric variables were equivalent between the two patient groups. The current data have not been disaggregated by sex, as the sample size was insufficient to support such sub-analyses. 

**Figure 1.**
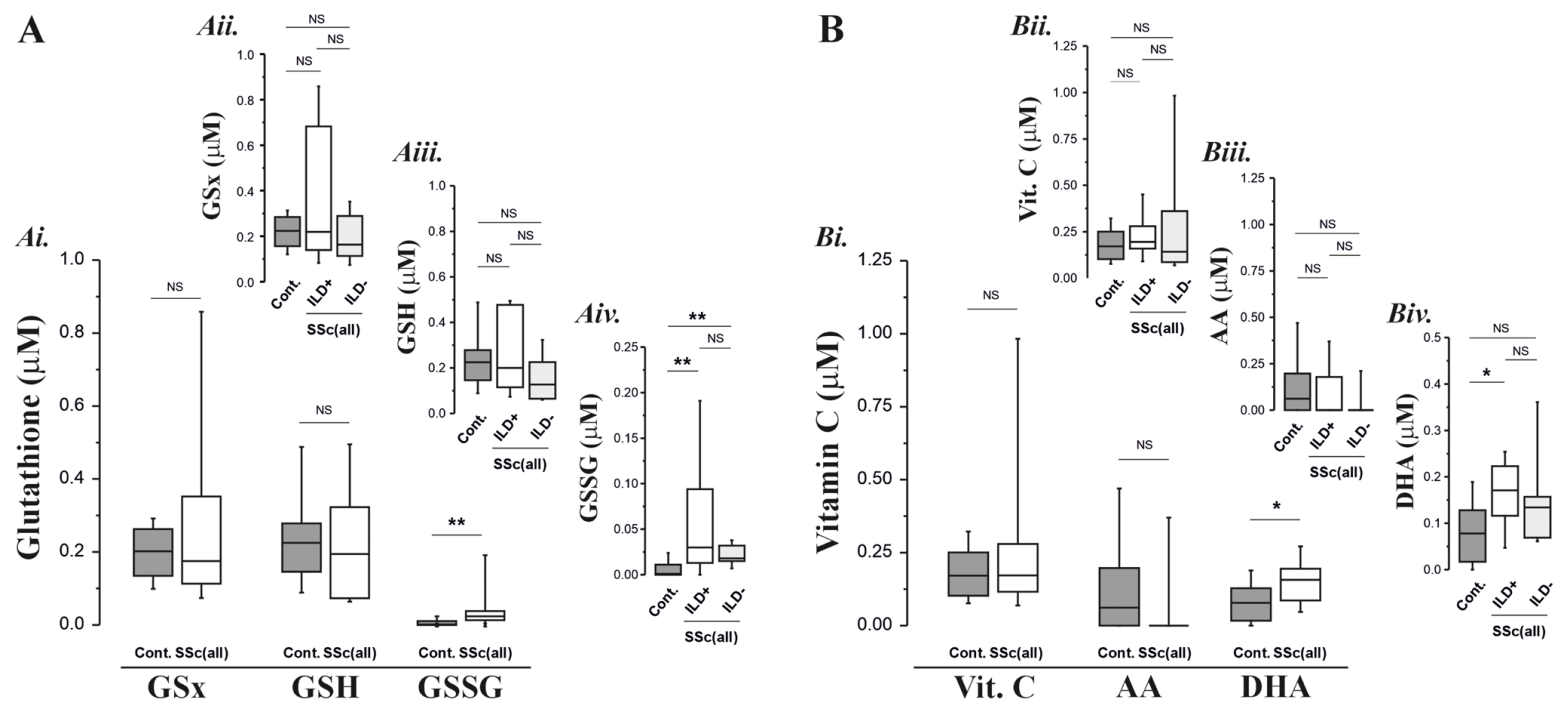
Antioxidant concentrations in lavage fluids from SSc patients and age-matched controls. **Panel A**: Total (GSx), reduced (GSH) and oxidised (GSSG) glutathione concentrations in BAL fluid from all SSc patients (both with and without ILD) and age-matched controls (Ai.). Panels Aii. – Aiv. illustrate the patterns of response in controls, SSc patients with ILD and without ILD (ILD+ vs. ILD-). **Panel B**: Vitamin C (Vit. C), ascorbate (AH 2) and dehydroascorbate (DHA) concentrations in BAL fluid obtained from all SSc patients (both with and without ILD) and age-matched controls. NS: not significantly different; * P<0.05, ** P<0.01, *** P<0.001.

We observed evidence of increased concentrations of GSSG (p=0.001, Figure 1, panel A) and DHA (p=0.04, Figure 1, panel B) in BAL fluid samples from the SSc patients compared to the age-matched controls, all consistent with the presence of oxidative stress. The increase in these oxidation products was seen in the absence of corresponding decreases in GSH or AA ( Figure 1) and without significant decreases in the concentration of RTLF glutathione ( Figure 1, panel A), vitamin C and ascorbate ( Figure 3, panel B). It was notable that where evidence of oxidative stress was apparent (specifically increased GSSG and DHA concentrations), this appeared to be a generic feature of SSc, irrespective of the presence of ILD ( **panels Aiv** and **Biv**). These responses were robust to the omission of patients who were smokers or taking oral corticosteroids.

**Figure 2.**
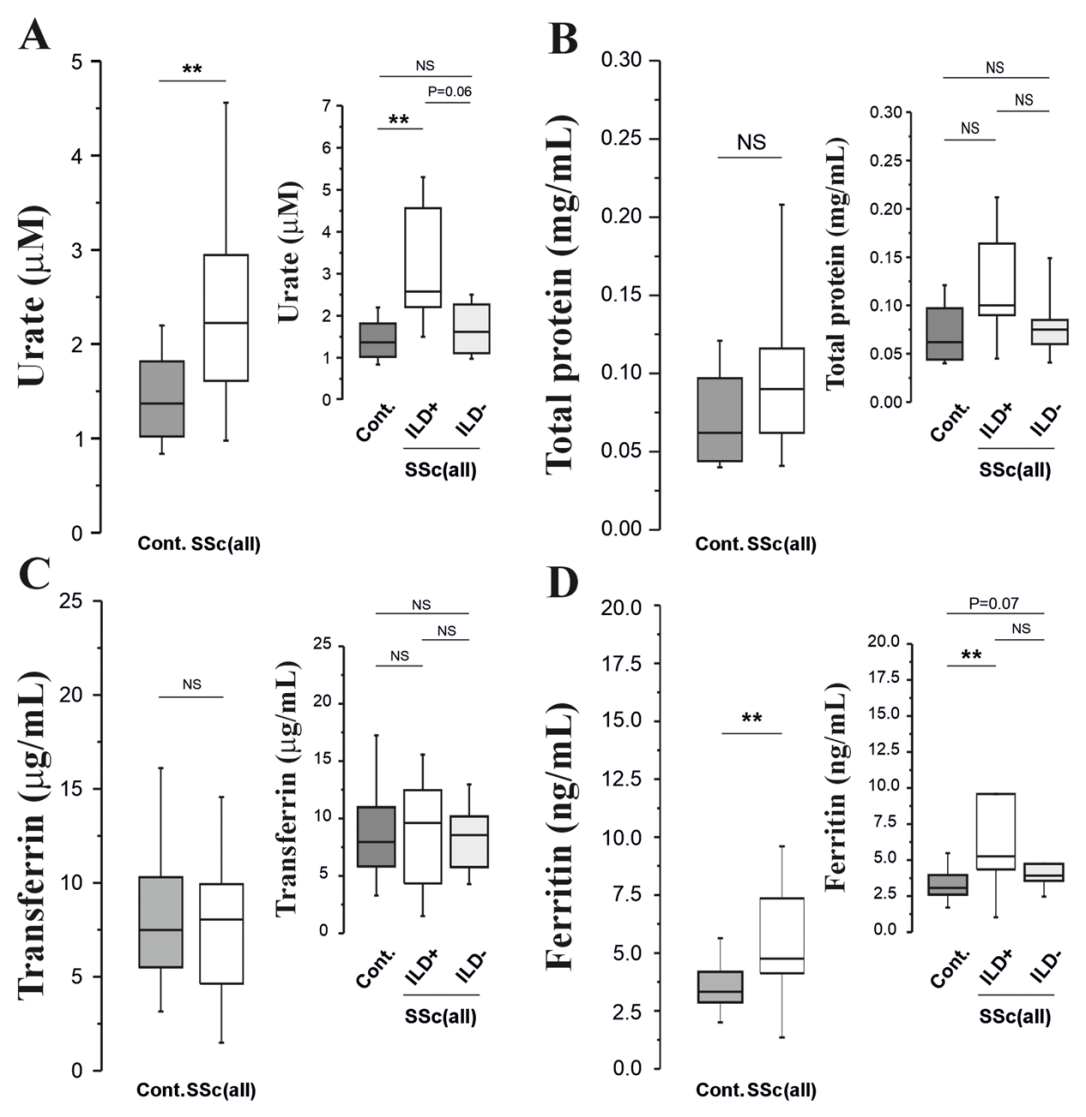
**Panels A–D**: Urate, total protein, transferrin and ferritin concentrations in BAL fluid obtained from all SSc patients (both with and without ILD) and age-matched controls.

Urate concentrations were significantly elevated in SSc patients (p=0.009, Figure 2, panel A), with some evidence that concentrations were elevated (P=0.06) in SSc-ILD patients compared with SSc patients without ILD ( Figure 2, panel A). Lavage total protein concentrations were also measured but did not differ between the groups ( Figure 2, panel B). Transferrin concentrations were also equivalent across all groups ( Figure 2, panel C), but ferritin concentrations were elevated in SSc patients (P=0.009, Figure 2, panel D). There was no significant difference between patients and controls with regard to protein carbonyl concentrations: (0.01 (0.00-0.04) *vs.* 0.01 (0.00-0.02) nM/mg protein.

**Figure 3.**
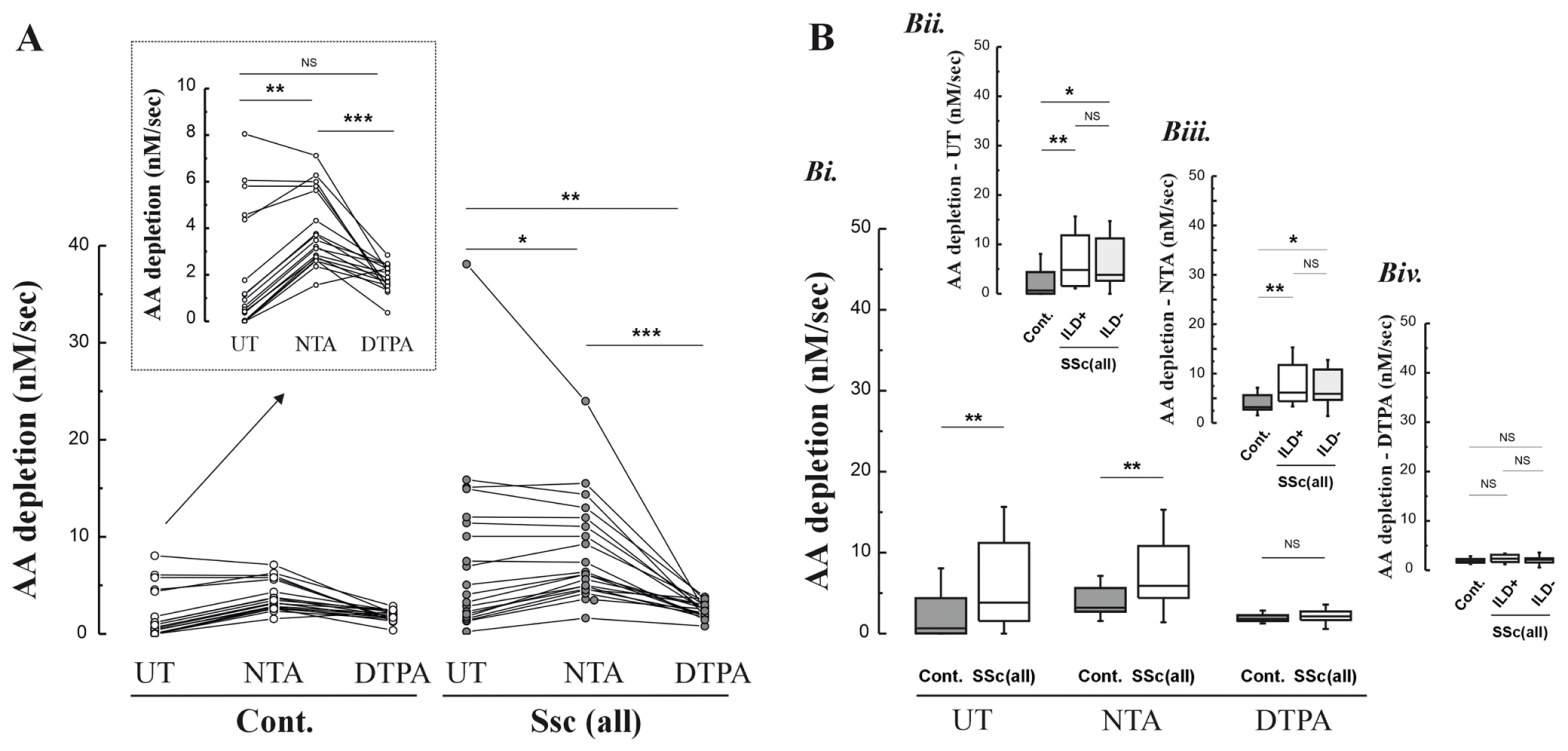
**Panel A**: Ascorbate (AA) oxidation rates in BAL with/without the addition of the chelators NTA and DTPA (200 μM). **Panel B**: Calculated AA depletion rates across chelation conditions, all SSc patients (both with and without ILD) with age-matched controls (Bi.), and for SSc patients with ILD and without ILD (ILD+ vs. ILD-) with age-matched controls (Bii. –Biv.).

**Figure 4.**
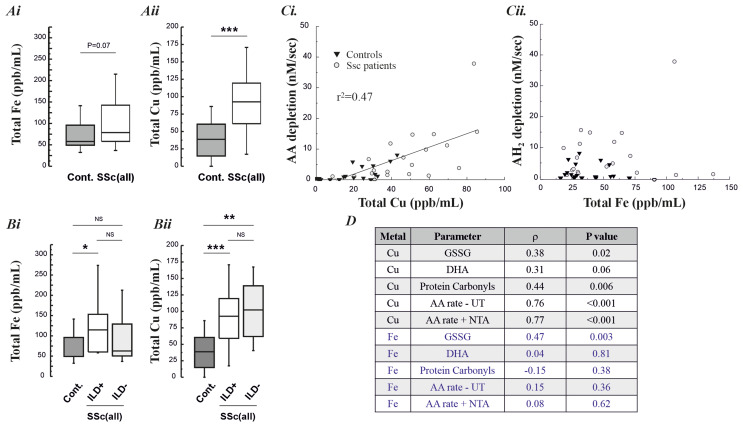
**Panel A–B**: Total BAL fluid Fe and Cu content, across all SSc patients (both with and without ILD) (Ai.-Aii.) and patients with and without ILD (Bi. and Bii.), with age-matched controls. **Panel C**: The relationship between the measured BAL Cu and Fe content and the observed rate of ascorbate depletion. **Panel D**: Associations between the concentrations of lavage fluid Fe and Cu with the measured oxidative damage markers and the pro-oxidant rate characteristics of the lavage, with and without the activation of non-transferrin bound Fe with NTA.

To investigate the basis for the elevated concentrations of GSSG and DHA in the BAL samples from the SSc patients, we investigated the pro-oxidant nature of the lavage returns using the rate of ascorbate oxidation following addition of 200 μM of AA. To elucidate the role of the redox active metals Cu and Fe, these experiments were also performed in the presence of DTPA, to quench the catalytic action of these metals, and NTA, which will bind to non-transferrin bound iron (NTBI), whilst permitting it to redox cycle ^32^. These data indicated that AA depletion rates were significantly increased in both control subjects and SSc patients following treatment of NTA, consistent with the presence of a small NTBI pool, but could be abolished by DTPA ( Figure 3, panel A). The lavage returns from SSC patients were significantly more oxidising than those from aged-matched controls, but consistent with the previous data there was no clear difference in rates between SSc patients with or without ILD ( Figure 3, panel B). Fe concentrations were not greater in lavage samples from the SSc patients compared with controls, p=0.07 (p=0.02 in subgroup analysis for SSc-ILD versus controls). By contrast, Cu was significantly elevated in both SSc patient groups ( Figure 4, panels Aii, Bii). When the Fe and Cu content of the lavage samples was related to the concentration of oxidation markers, or the pro-oxidant nature of the samples, only Cu demonstrated a robust association ( Figure 4, panels D). BAL Cu content was significantly associated with the measured concentrations of GSSG (r=0.38, P=0.02) and protein carbonyls (r=0.44, P=0.006), and trended toward significance for DHA (r=0.31, P=0.06) in the lavage samples. The lavage Cu content was also significantly associated with the observed ascorbate depletion rate (r=0.76, P<0.001). By contrast, lavage Fe was only associated with BAL GSSG concentrations (r=0.47, P=0.003).

## Discussion

SSc is a rare autoimmune condition characterised by fibrosis and vascular changes of the skin and internal organs, mainly affecting young and middle-aged women. SSc-ILD is a common manifestation of SSc that can lead to progressive fibrosis of the lungs and remains the main cause of mortality in this patient group. Treatment options are still limited, which is why it remains imperative that the disease mechanisms be further clarified to allow for improved therapeutical options as well as to identify relevant biomarkers that can be utilised for disease detection and monitoring.

There is ample *in vivo* and *in vitro* evidence of increased levels of oxidative stress during all stages of SSc disease development ^1, 33^. Increased levels of ROS and oxidative stress have been found to play a direct role in the onset and progression of key processes in disease development, such as perfusion-reperfusion injury, and ligand-mediated receptor activation by cytokines and growth factors can increase ROS levels further ^34, 35^. Some evidence also points to a possible connection between fibrosis progression in SSc-ILD and endothelial-to-mesenchymal-transition (EndMT), which is also found in other fibrotic lung diseases ^33^. There is also extensive literature to support oxidative stress as a key step in the pathophysiology of other interstitial lung diseases ^33^.

To our knowledge, no data are available on the role of catalytic metals in the airways of patients with SSc and/or SSc-ILD. Looking outside of the airways, available data are diverging on whether systemic copper dysregulation is present in this patient group ^23^. Considering other diseases that share pathophysiological similarities with SSc, having either inflammatory and/or autoimmune properties, there is a limited number of small studies performed on patients with diabetes mellitus and rheumatoid arthritis, with data suggesting a possible role of copper dysregulation in disease progression, although the significance of that role is unclear and further studies are needed to confirm the results ^36, 37^. When comparing SSc-ILD to other interstitial lung diseases, some data suggest altered BAL trace metal levels in IPF patients compared to controls ^24^.

In the current study, we investigated whether metal dysregulation, specifically of iron and copper, was present in the respiratory tract lining fluid (RTLF) of patients with SSc and SSc-ILD, and whether this dysregulation contributed to oxidative stress within the RTLF, using BAL.

These data confirm the presence of oxidative stress in the airways of SSc patients and, suggesting a potential role for dysregulated iron and copper homeostasis at the air-lung interface in disease progression. This is a notable observation as metal chelation therapies are available and have demonstrated clinical utility in various disease contexts ^38^.

However, our findings regarding oxidative stress markers and catalytic metals in SSc patients with and without ILD were not as straightforward as anticipated. While previous work in IPF patients showed elevated oxidative stress markers compared to controls, we did not observe a similarly pronounced difference between the SSc subgroups ^39^.

A closer examination of individual markers, such as urate and ferritin, reveals a more nuanced picture. The overall distribution of GSSG, ferritin, urate and DHA levels appears similar across groups, with significant differences between SSc-ILD patients and controls for urate and ferritin. Specifically, urate levels in SSc-ILD patients trended towards a significant difference compared to SSc patients without ILD (p=0.06; Figure 2, panel A). This borderline significance may be attributable to the limited sample size, suggesting that a larger study could reveal a clearer distinction. Interpreting DHA levels is further complicated by a lack of baseline BAL data in SSc populations and limited information on the correlation between blood and BAL glutathione levels.

Another important consideration is the distinct behavior of these redox markers. AA levels in the airways are dependent on dietary intake and transport from the blood. While mechanisms for the conversion of DHA back to AA have been described, a corresponding recycling mechanism within the RTLF has been postulated but not yet identified. Therefore, assuming uniform behavior across all oxidative stress markers would be overly simplistic, given their varying mechanisms of oxidation, recycling and replenishment within the RTLF.

Finally, the timing of bronchoscopy in relation to individual patient disease course may have influenced our findings. It is conceivable that SSc patients without ILD at the time of bronchoscopy had more active inflammation and, thus, more pronounced differences in DHA/GSSG levels compared to controls. Conversely, SSc-ILD patients at the time of bronchoscopy may have had a more indolent inflammatory state, having already developed established ILD and Sjögren’s syndrome (SS). These preliminary findings warrant further investigation into the role of redox-active metals in SSc pathogenesis.

As can be seen in the study results, the SSc-ILD group had older individuals with more severe disease compared to the individuals in the SSc group without ILD. Yet no difference was found between these two groups. It is possible that the lack of difference between the groups could at least in part be accounted for by disease duration. Due to its cross-sectional design, the study was not able to follow changes over time, which would be useful when trying to disentangle any association between fibrosis development, disease activity and disease duration. The study population included patients with both early and late disease, and the number of patients was insufficient to perform a subgroup analysis focusing on disease duration. Whilst SSc-ILD was traditionally assumed to be a slowly progressing disease, more recent data have shown that the clinical trajectories are divergent, with older age, male sex and smoking at the time of diagnosis being associated with a more rapidly progressive disease ^40^. Thus, subgroup analysis exploring the effect of age, sex and/or smoking is warranted.

There is also a treatment factor to consider when evaluating the current findings. Of the 20 SSc patients that were included in this study, six were already being treated with corticosteroids at the time of the bronchoscopy. Corticosteroids, when given in monotherapy to SSc-ILD patients, have been shown to stabilise lung function over time, though at the cost of side effects. Therefore, corticosteroids are most often given in combination with other immunosuppressive, biological and/or antifibrotic agents in order to minimise the total corticosteroid burden ^41^. We are not able to disentangle whether the current findings have been impacted by the effect of corticosteroids, possibly contributing to an evening out of the study findings between the groups of SSc patients with and without ILD. Furthermore, patients on corticosteroid treatment at the time of bronchoscopy might have been those with the highest risk of progressive fibrosis, with signs of active disease at the time of the initial presentation and, if so, that was the reason why they had been put on corticosteroid treatment early. This possibility makes the impact of corticosteroid treatment difficult to evaluate.

While limited data exist regarding the role of iron and copper in other fibrotic lung diseases, comparisons with these conditions may offer valuable insights into SSc-ILD. However, caution is warranted due to potentially distinct etiologies and risk factors. For instance, the genetic underpinnings of SSc-ILD differ from those of IPF, where the MUC5B gene plays a significant role in disease development ^42^. SSc susceptibility is associated with several human leukocyte antigen (HLA) and non-HLA genes, primarily related to immunological pathways, though their individual effects on disease development appear modest ^43^. Notably, the number of identified susceptibility genes for SSc-ILD is considerably smaller than for SSc. Despite these differences, shared mechanisms may be operative. Oxidative stress, including Nrf2-pathways that regulate the antioxidative network, has been implicated in IPF pathophysiology ^39^. Interestingly, pirfenidone, a recognized therapeutic agent for IPF, exhibits antioxidative properties, and interacts with iron ^44^.

This study's primary limitation is the small sample size of SSc patients, both with and without ILD. Recruiting sufficient patient numbers for a single-center study of this rare disease proved challenging. Consequently, we were unable to explore the influence of biological sex on the measured endpoints, and the limited sample size may have hampered our ability to detect more pronounced differences between patients with and without ILD. Larger, multi-center studies are needed to address these questions and enable a more thorough analysis of potential disease risk modifiers. Furthermore, the study lacked certain relevant background information, specifically regarding smoking history and occupational exposures, which would have strengthened the analysis.

The patients had been carefully diagnosed by an experienced rheumatologist. It is however worth noting that when comparing different studies on SSc-ILD, the diagnostic criteria have been revised over time, which is reflected in older studies having slightly different study populations.

In the current study, patients have been diagnosed using older criteria (1980 ARA) ^45^. Likewise, the classification criteria for SS have been revised over time and for a long time universal terminology was lacking. The selection of patients for the current study was based on available criteria at the time of inclusion and these criteria have subsequently altered, which inhibits some of the generalisability of the study findings. In this study, the group with SSc and ILD group has a higher proportion of patients with SS than the group with SSc without ILD. While this represents a significant bias, it is worth noting that we do not have data on the ratio between primary and secondary SS nor on sicca symptoms nor on treatment with immunosuppressants. It is likely that this group of patients would have been composed and treated differently if the study had been carried out today, and the incidence of SS in the SSc-ILD group would likely have been lower.

Nevertheless, these data confirm the presence of oxidative stress in the airways of patients with SSc and, for the first time, suggest that an underlying defect in iron and copper homeostasis at the air-lung interface may play a role in the disease progression. Despite this, we observed no clear difference in the concentration of oxidation markers or catalytic metals between SSc patients with and without ILD, though we see trends supporting elevated urate and ferritin concentrations in SSc patients with ILD that warrant further investigation.

## Data Availability

The data underlying this article cannot be shared publicly for the privacy of individuals that participated in the study, due to limitations imposed by Swedish jurisdiction on storage and sharing of personal data. Aggregated, fully anonymised data may be shared in line with European GDPR regulations on reasonable request to the corresponding author.
