# Supplementary material for: Respiratory tract lining fluid copper content contributes to pulmonary oxidative stress in patients with systemic sclerosis
Source: Wellcome Open Res. 2025 Mar 4;9:139. Originally published 2024 Mar 8. [Version 2] doi: 10.12688/wellcomeopenres.20080.2 (PMC11923536; doi:10.12688/wellcomeopenres.20080.2)
Supplement: Peer review history

# Reviewer report: Reviewer response for version 2

**DOI:** 10.21956/wellcomeopenres.25973.r120116

**Published:** 2025-03-19

**Authors:** Bettina Schock

**Affiliations:** 1 Queen's University Belfast, Belfast, Northern Ireland, UK

The authors appropriately addressed all queries and comments

Is the work clearly and accurately presented and does it cite the current literature?

Yes

If applicable, is the statistical analysis and its interpretation appropriate?

Yes

Are all the source data underlying the results available to ensure full reproducibility?

Yes

Is the study design appropriate and is the work technically sound?

Yes

Are the conclusions drawn adequately supported by the results?

Partly

Are sufficient details of methods and analysis provided to allow replication by others?

Yes

Reviewer Expertise:

ILD including SSc-ILD

I confirm that I have read this submission and believe that I have an appropriate level of expertise to confirm that it is of an acceptable scientific standard.

# Reviewer report: Reviewer response for version 2

**DOI:** 10.21956/wellcomeopenres.25973.r120115

**Published:** 2025-03-06

**Authors:** Hanlin Yin

**Affiliations:** 1 Shanghai Jiao Tong University School of Medicine, Shanghai,, China

The revised manuscript has addressed my concerns satisfactorily.

Is the work clearly and accurately presented and does it cite the current literature?

Yes

If applicable, is the statistical analysis and its interpretation appropriate?

Yes

Are all the source data underlying the results available to ensure full reproducibility?

Yes

Is the study design appropriate and is the work technically sound?

Yes

Are the conclusions drawn adequately supported by the results?

Yes

Are sufficient details of methods and analysis provided to allow replication by others?

Yes

Reviewer Expertise:

SSc included SSc-ILD

I confirm that I have read this submission and believe that I have an appropriate level of expertise to confirm that it is of an acceptable scientific standard.

# Reviewer report: Reviewer response for version 1

**DOI:** 10.21956/wellcomeopenres.22238.r85331

**Published:** 2024-06-26

**Authors:** Hanlin Yin

**Affiliations:** 1 Shanghai Jiao Tong University School of Medicine, Shanghai,, China

Manuscript Review: Andreas Frølich et al.

 The manuscript by Andreas Frølich et al. describes the levels of metal ions and the degree of oxidative stress in alveolar lavage fluid from patients with systemic sclerosis.The whole text is well structured with clear diagrams.

 This is the first article to demonstrate a correlation between metal ions in alveolar lavage fluid and oxidative stress in the lungs of patients with systemic sclerosis,which is highly novel.

 Nevertheless, when the text discusses the strong link between oxidative stress and systemic sclerosis, it would be beneficial to cite more recent authoritative sources  in order to corroborate the authors' views. For example, the following sources could be referenced: (Michael Hughes et al. 2015) [Ref 1] and (Svegliati S et al. 2018) [Ref 2].

 Table I demonstrates that the group of SSc patients with interstitial lung disease included in the text exhibited a markedly higher prevalence of combined Sjögren's syndrome than the group without interstitial lung disease. This represents a significant bias.

 Figure 2 is not presented in an optimal manner and the scale of the graph could be adjusted accordingly.

 In conclusion, the results of this article are novel but require further modification.

Is the work clearly and accurately presented and does it cite the current literature?

Yes

If applicable, is the statistical analysis and its interpretation appropriate?

Yes

Are all the source data underlying the results available to ensure full reproducibility?

Yes

Is the study design appropriate and is the work technically sound?

Yes

Are the conclusions drawn adequately supported by the results?

Yes

Are sufficient details of methods and analysis provided to allow replication by others?

Yes

Reviewer Expertise:

SSc included SSc-ILD

I confirm that I have read this submission and believe that I have an appropriate level of expertise to confirm that it is of an acceptable scientific standard, however I have significant reservations, as outlined above.

## References

[1] : Abnormalities of selenium but not of copper homeostasis may drive tissue fibrosis in patients with systemic sclerosis. *Rheumatology* .2015;54(4) : 10.1093/rheumatology/keu470 747-748 10.1093/rheumatology/keu470 25602061

[2] : NADPH oxidase, oxidative stress and fibrosis in systemic sclerosis. *Free Radic Biol Med* .2018;125: 10.1016/j.freeradbiomed.2018.04.554 90-97 10.1016/j.freeradbiomed.2018.04.554 29694853

# Response

**Published:** 2025-02-14

**Authors:** Andreas Froelich

**Affiliations:** Department of Public Health and Clinical Medicine, Umeå University, Umeå, Västerbotten County, Sweden

On behalf of the team of authors I would like to thank you for reviewing our work and giving us your expert opinion. We have used your valuable feedback to improve our manuscript further.  

 1.Nevertheless, when the text discusses the strong link between oxidative stress and systemic sclerosis, it would be beneficial to cite more recent authoritative sources  in order to corroborate the authors' views. For example, the following sources could be referenced: (Michael Hughes et al. 2015) [Ref 1] and (Svegliati S et al. 2018) [Ref 2].

**Response: **We agree. More recent authoritative sources are needed to strengthen our views. We have now added both publications to the text as references.  

 2. Table I demonstrates that the group of SSc patients with interstitial lung disease included in the text exhibited a markedly higher prevalence of combined Sjögren's syndrome than the group without interstitial lung disease. This represents a significant bias.

**Response:** Yes, it is a significant bias. After this was pointed out to us, we consulted a senior rheumatologist and made adjustments to the text.  We consider this to be a limitation of the study and in the discussion section we argue how the data may be viewed in light of this limitation.   Figure 2 is not presented in an optimal manner and the scale of the graph could be adjusted accordingly.

**Response:** We fully agree that is not optimal. We have considered an alternative, which would be a re-adjustment of the scale. As the study groups were small and the results when comparing groups not generally clear-cut, we considered that a readjustment of the scale would overemphasise whatever positive results we got, and we wanted to reduce that risk. That is why we at the end decided to keep the figures as it stands, even though we agree that it is not optimal.

# Reviewer report: Reviewer response for version 1

**DOI:** 10.21956/wellcomeopenres.22238.r78601

**Published:** 2024-05-06

**Authors:** Bettina Schock

**Affiliations:** 1 Queen's University Belfast, Belfast, Northern Ireland, UK

Manuscript Review: Andreas Frølich et al.

 The manuscript by Andreas Frølich et al. describes the metal ion content in the respiratory tract lining fluid from patients with Systemic Sclerosis.

 The manuscript Is well written and well-structured.

 The existence of oxidative stress in the pathogenesis of systemic sclerosis due to inflammation, autoimmunity and fibrosis has been described before (Doridot et al. 2019) [Ref 1], however, this is the first report of oxidative stress and reactive oxidant species in bronchoalveolar lavage fluid in SSc and SSc-ILD.

 The introduction should more clearly differentiate between SSc and SSc-ILD when describing findings in the literature. It is not clear from the introduction if the focus of this work is on oxidative stress in SSc or in SSc-ILD or both.

 Although it is mentioned that there is 'ample evidence’ of oxidative stress in SSc and SSc-ILD pathology, the paper by e.g., (Doridot et al. 2019) [Ref 1] and references for ILD or IPF should be discussed (e.g., Bast et al. 2021) [Ref 2] / Estornut et al. 2021[Ref 3] should be included.

 Combining all figures for Figure 2A (the same for Fig 2B and subsequent figures 3A-D and 4B). The graphs will be much easier to read and repetition of controls results can be avoided. E.g., each graph could have data for Control, all SSc, SSc without ILD and SSc-with ILD

 Figure 5, panel Ci and Cii, could these correlations include a different coding for SSC (without and SSc with ILD)?

 The described results are novel and show for some of the investigated parameters an increase /decrease in SSc-ILD compared to controls, suggesting additional pulmonary oxidative stress. In particular is appears that copper (Cu) induced oxidative stress markers are more affected than ion (Fe) induced makers (Pannel 5D). However, there is a lack of modified oxidative stress measures when RTLF from SSc patients is compared to those with SSc-ILD. Here, only DHA levels are significantly different. Considering that RTLF fluid and not serum/plasma was investigated, this needs a stronger discussion (including the lack of differences between SSc and SSc-ILD and the potential interpretation of the DHA levels in SSc versus SSc-ILD.

Is the work clearly and accurately presented and does it cite the current literature?

Yes

If applicable, is the statistical analysis and its interpretation appropriate?

Yes

Are all the source data underlying the results available to ensure full reproducibility?

Yes

Is the study design appropriate and is the work technically sound?

Yes

Are the conclusions drawn adequately supported by the results?

Partly

Are sufficient details of methods and analysis provided to allow replication by others?

Yes

Reviewer Expertise:

ILD including SSc-ILD

I confirm that I have read this submission and believe that I have an appropriate level of expertise to confirm that it is of an acceptable scientific standard, however I have significant reservations, as outlined above.

## References

[1] : Implication of oxidative stress in the pathogenesis of systemic sclerosis via inflammation, autoimmunity and fibrosis. *Redox Biol* .2019;25: 10.1016/j.redox.2019.101122 101122 10.1016/j.redox.2019.101122 30737171 PMC6859527

[2] : Oxidative stress and antioxidants in interstitial lung disease. *Curr Opin Pulm Med* .2010;16(5) : 10.1097/MCP.0b013e32833c645d 516-20 10.1097/MCP.0b013e32833c645d 20592594

[3] : Targeting Oxidative Stress as a Therapeutic Approach for Idiopathic Pulmonary Fibrosis. *Front Pharmacol* .2021;12: 10.3389/fphar.2021.794997 794997 10.3389/fphar.2021.794997 35126133 PMC8815729

# Response

**Published:** 2025-02-14

**Authors:** Andreas Froelich

**Affiliations:** Department of Public Health and Clinical Medicine, Umeå University, Umeå, Västerbotten County, Sweden

On behalf of the team of authors I would like to thank you for your expert opinion. Your valuable feedback will improve the manuscript further. Hopefully, the following responses address your concerns and questions.

 1. The introduction should more clearly differentiate between SSc and SSc-ILD when describing findings in the literature. It is not clear from the introduction if the focus of this work is on oxidative stress in SSc or in SSc-ILD or both.

**Response:** Thank you for this observation. We agree that the introduction needs a clearer distinction between SSc with and without ILD, especially when relating to findings in the areas of oxidative stress and metals. As a consequence, we have expanded the introduction section, aiming to relating relevant findings to SSc first and thereafter to SSc-ILD, where applicable.  

 2. Although it is mentioned that there is 'ample evidence’ of oxidative stress in SSc and SSc-ILD pathology, the paper by e.g., (Doridot et al. 2019) [Ref 1] and references for ILD or IPF should be discussed (e.g., Bast et al. 2021) [Ref 2] / Estornut et al. 2021[Ref 3] should be included. 

**Response:** We agree that the paper should be supported by these more recent and authoritative publications. We have added the publications you suggested and a few others to the manuscript. Following your suggestion, we have also added reference to other types of ILD.

 3. Combining all figures for Figure 2A (the same for Fig 2B and subsequent figures 3A-D and 4B). The graphs will be much easier to read and repetition of controls results can be avoided. E.g., each graph could have data for Control, all SSc, SSc without ILD and SSc-with ILD Figure 5, panel Ci and Cii, could these correlations include a different coding for SSC (without and SSc with ILD)?

**Response:** Thank you for your observation and we agree that the figure can be hard to read. The problem that we see with combining the figures is that the statistical methodology used in the current study is composed of two distinct separate steps. We wanted to avoid any possible misconception of the groups being compared in one step. By mixing these two steps in one combined graph runs that risk. To increase readability and reduce the risk of ambiguity, we have consequently changed the figure legends. Hopefully, this change will make it easier to distinguish the subgroups from each other and make it unambiguous that the “first part” of the figure is a comparison between all SSc patients (both with ILD and without ILD) versus controls.

 4. However, there is a lack of modified oxidative stress measures when RTLF from SSc patients is compared to those with SSc-ILD. Here, only DHA levels are significantly different. Considering that RTLF fluid and not serum/plasma was investigated, this needs a stronger discussion (including the lack of differences between SSc and SSc-ILD and the potential interpretation of the DHA levels in SSc versus SSc-ILD.

**Response:** Thank you for your important point. We agree that based on previous BAL data from ILD -patients on oxidative stress, it was fair to assume that the current study results would show a more clear-cut difference between SSc with and without ILD. Reflecting this issue, we have thus expanded the discussion section, allowing room for possible explanations.
